# Raw Chickpea (*Cicer arietinum* L.) as a Substitute of Soybean Meal in Compound Feed for Broiler Chickens: Effects on Growth Performance, Lipid Metabolism, Fatty Acid Profile, Antioxidant Status, and Dietary Value of Muscles

**DOI:** 10.3390/ani11123367

**Published:** 2021-11-24

**Authors:** Anna Danek-Majewska, Małgorzata Kwiecień, Anna Winiarska-Mieczan, Małgorzata Haliniarz, Agata Bielak

**Affiliations:** 1 Institute of Animal Nutrition and Bromatology, University of Life Sciences, Akademicka Street 13, 20-950 Lublin, Poland; anna.majewska@up.lublin.pl (A.D.-M.); anna.mieczan@up.lublin.pl (A.W.-M.); agata.bielak@up.lublin.pl (A.B.); 2 Department of Herbology and Plant Cultivation Techniques, University of Life Sciences, Akademicka Street 13, 20-950 Lublin, Poland; malgorzata.haliniarz@up.lublin.pl

**Keywords:** chickpea, broiler chickens, antioxidant status, fatty acid, meat quality

## Abstract

**Simple Summary:**

Defatted soybean meal is the basic source of protein in poultry diet. Therefore, researchers are searching for an alternative source of vegetable protein derived from native raw materials. The present results were obtained in an experiment consisting in the use of soybean meal in the diet for broiler chickens, or the replacement of 50% of soybean meal protein with raw chickpea seed protein. The impact of the substitution on the poultry production process and on the dietary value of poultry meat was assessed.

**Abstract:**

The aim of this study was to determine the effect of substitution of 50% of soybean meal protein with 310–350 g/kg diet of raw chickpea seed protein on the chemical composition, fatty acid profile, dietary value, and antioxidant status of breast and thigh muscles, as well as the antioxidant status of blood serum, in Ross 308 male broilers. In the 42-day experiment, one-day-old male broiler chicks were assigned to two nutritional groups (*n* = 100 in each, 20 birds in each group, and 5 replications). In the control group, 100% of protein in the feed was derived from soybean meal. In the experimental group, 310–350 g/kg protein from raw chickpea seeds was introduced. Data with a normal distribution were analyzed using the Student *t*-test, and the relationships between the traits were assessed with the use of Pearson’s correlation coefficients. *p* < 0.05 was considered statistically significant. The replacement with chickpea protein did not exert an impact on the final body weight, feed consumption, and feed conversion ratio compared to the control group. However, it induced changes in the color of the breast muscles (increased L* and b* values), and reduced the cholesterol content. The addition of chickpea seeds improved the fatty acid profile, mainly in the breast muscle. A decrease in the total SFA content and a higher level of unsaturated fatty acids (UFA), UFAs/saturated fatty acids (SFAs), polyunsaturated fatty acids (PUFAs), omega-3, and omega-6 were observed in the experimental group. Additionally, the chickpea-supplemented group exhibited better values of meat quality indicators (atherogenic index-AI; thrombogenic index–TI, ratio of saturated fatty acids to unsaturated fatty acids-S/P, *n*-6/*n*-3, hypocholesterolemic/Hypercholesterolemic ratio-h/H). It can be concluded that raw chickpea seeds are a good source of protein in broiler chicken nutrition, and can replace the traditionally used protein source (soybean meal), simultaneously exerting a positive effect on the dietary value of poultry meat and an expected enhancing impact on consumer health.

## 1. Introduction

In addition to genotype, age, sex, and housing systems, nutrition is one of the most important determinants of the quality of meat. Consumers are paying more attention to the safety and quality of poultry products. The consumption of poultry products shows an upward trend [1], since poultry meat is cheaper than other types of meat. It is also easy to cook and simple to prepare. Poultry meat provides easily digestible protein, and has a low content of cholesterol and fat. It is rich in polyunsaturated fatty acids (PUFAs), especially omega *n*-3 fatty acids, which are generally consumed by humans in insufficient amounts, due to the lack of omega *n*-3-rich products [2]. On the one hand, PUFAs are necessary for the proper functioning of an organism, as these acids help to prevent or alleviate many civilization diseases, including autoimmune diseases, heart attack, coronary artery disease, stroke, or some neoplasms [3]. On the other hand, meat that is rich in *n*-3 and *n*-6 acids is especially susceptible to oxidative rancidity, which deteriorates its sensory value, and causes changes in the physical properties of fresh and thawed meat [4,5]. Lipid oxidation is accompanied by generation of compounds that may exert a negative effect on consumer health due to their mutagenic, carcinogenic, and cytotoxic properties [6].

The basic high-protein feed used in poultry diets is soybean meal (SBM) [7], which has an optimal protein composition, and low fiber content. Unfortunately, the constant rise in SBM prices diminishes the already low profitability of production of all poultry species. An additional problem is the intended introduction of a ban on the import and distribution of feed containing genetically modified (GM) plants. Consumers often express concern over the potential threat posed by products obtained from animals receiving GM feed. The deficit of plant proteins is reported in the entire European Community, and various actions are undertaken to mitigate the problem. Currently, Poland imports over 75–85% of feed protein, and only approximately 15–25% is produced in the country [8]. In Poland, the undertaken measures are targeted at an approximately 50% reduction of the import of feed protein (SBM) through enhancement of the biological and functional value of vegetable protein derived from native raw materials. A possibility of partial substitution of SBM is ascribed to the use of legume seeds. In addition to raw or processed pea, lupine, or horse bean seeds [9,10,11], which have been investigated to assess their suitability for use in animal nutrition, the chickpea (*Cicer arietinum* L.) [12,13,14,15] deserves attention. The plant is not very common in Poland, but is one of the most important legumes in the world [16]. On the one hand, chickpea can secure protein reserves in the country; on the other hand, it can ensure availability of animal products that meet customer expectations, i.e., food produced with the use of domestic raw materials without the addition of GM plants. Chickpea seeds have a high nutritional value, high protein content (16.7–30.6%) [17], and ME (11.5–13.2 MJ/kg DM) [18]. Moreover, they are a source of minerals (Ca, P, Mg, Fe, K), vitamins B, and fiber [19]. Similar to other legumes, chickpea seeds contain many anti-nutritional factors (ANF), such as proteases and amylase inhibitors, as well as lectins, polyphenols, and oligosaccharides, which impair nutrient absorption and exert a detrimental effect on animal growth and health [20]. However, in comparison with other legumes, chickpea seeds contain relatively small amounts of trypsin and chymotrypsin inhibitors, hence, they cause fewer health problems in poultry. As reported by Bampidis et al. [17], raw chickpea seeds (CPR) can be added to poultry diets at a level of 15–20% to achieve a positive effect on poultry growth and egg production. A higher level of supplementation with chickpea seeds in the diet for broilers has been found to exert a negative effect on weight gain, feed intake, and feed conversion rates [21].

To the best of our knowledge, there are only few studies [12,13,14,15] focused on assessing the effect of CPR-supplemented nutrition on broiler chickens. We hypothesized that the basic source of protein in the diet may have an impact on meat resistance to oxidative processes, the fatty acid profile, and the lipid and oxidative profile in chicken blood. Therefore, this study investigated the effect of replacement of 50% of soybean meal protein with 310–350 g/kg diet of raw chickpea seed protein on the chemical composition, fatty acid profile, dietary value, and antioxidant status of breast and thigh muscles, as well as the antioxidant status in the serum, of Ross 308 male broilers.

## 2. Materials and Methods

### 2.1. Chickpea Seeds, Experimental Design, and Management

Unprocessed large, round, light cream-colored chickpea seeds (*Cicer arietinum* L.) cv. Sanford (morphological kabuki type) were purchased from a certified seed material seller (Lublin, Poland). This variety is cultivated in the southern and eastern parts of Poland. Its yield is estimated at 3.0 t⋅ha^−1^. The seeds used in the study were harvested in 2015.

The content of dry matter and essential nutrients in the chickpea seed samples (250 g of seeds) was determined according to the AOAC [22] standard procedures, as described in Section 2.3.1.

All procedures used during the research were approved by the Local Ethics Committee for Animal Testing at the University of Life Sciences in Lublin, Poland (Resolution No. 33/2015 of 26 May 2015). The study involved Ross 308 broiler cocks reared to 42 days of age on a poultry farm (Sędziszów, Małopolskie Province, Poland). One-day-old male broiler chicks (initial weight 38.8 ± 1.93 g) were randomly assigned to two groups (*n* = 100 in each group, and kept in 5 cages of 20). The initial temperature of 32 °C in the experimental room was gradually decreased during chick growth until week 4 of the experiment, then reaching 22 °C [23]. A three-phase nutrition scheme was applied: starter (0 to 21 days), grower (21 to 35 days), and finisher (35 to 42 days). The starter diet was administered in the crumble form, and the grower and finisher diets were granulated. Until 21 days of age (starter period), the male broilers in the control and experimental groups received diets composed of cereal meal (wheat and maize) and SBM. From rearing day 22, the chicks were fed in accordance with the methodological design of the experiment (Table 1). The source of protein in the mixture was the experimental factor. Our previous research [12,13,14,15] shows that CPR may be a promising substitute for SMB protein in broiler nutrition, in terms of animal welfare associated with the development of the skeletal system and structural changes in muscle tissue. Replacement of SBM as the basic protein in the diet with the CPR protein exerted a positive effect on the development of the skeletal system, improving overall bone development, bone strength, and spongy bone microarchitecture. Additionally, an interesting result of our research was the change in the level of collagen denaturation energy, as significantly greater changes in enthalpy were observed in the CPR group. This suggests that the tendons of birds from this group probably had morphologically denser collagen bundles than the tendons of birds from the SMB-receiving group. The level of the SBM CPR protein replacement was selected after analysis of results reported in the literature. The review showed that partial replacement (75, 120, 180, 240, 300, 360, 450, and 480 g/kg diets) of SBM protein with CPR raw seed protein in the diets for male broilers gave inconclusive results [21,24,25,26]. The introduction of partial SBM CPR replacement in the above-mentioned studies started on the first day of the chicks’ lives. Due to the presence of anti-nutritive substances, we decided to use raw chickpea seeds only in the grower and finisher stages, at 50% of the protein level from both feeds. SBM was the source of protein (100%) in the grower/finisher mixtures of the control group, whereas the grower and finisher diets of the experimental group contained 310–350 g/kg on diet of CPR-derived protein instead of 50% of SBM protein. The chemical composition of the former component is shown in Table 2. All diets were isoproteinous, isoenergetic, and balanced, as recommended by the nutritional guidelines for broiler chickens [23]. The birds had constant access to food and water. In accordance with the Aviagen recommendations [27], the 0- to 7-day-old chicks were kept at 23 h of light and 1 h of darkness. From day 8, the period of darkness was approximately 5 h. Until the seventh day of rearing, the light intensity was 35 lux; then, it was reduced to 5–10 lux from the eight day. During the dark period, the light intensity was below 0.4 lux.

### 2.2. Experimental Measurements

The male broilers were not fed 10 h before slaughter, but had constant access to water. Before slaughter in the morning on rearing day 42, the male broilers were weighed, and 10 birds with body weight representative for each group were selected [29]. The body weight gain (BWG) and feed conversion ratio (FCR) were calculated for the grower and finisher periods. Chick mortality was monitored every day. The weight of dead male broilers was included in the calculation of feed intake (FI) and FCR. After weighing, the male broilers were stunned electrically and slaughtered by decapitation. After slaughter, a simplified slaughter analysis was performed [30], during which the breast and thigh muscles were collected and weighed. The skin was separated from the muscles, which were then packed into single tightly sealed plastic bags and kept frozen at −20 °C until chemical analyses.

### 2.3. Sample Collection and Chemical Analyses

#### 2.3.1. Chickpea, Diets and Muscles

The raw chickpea seeds were analyzed to determine the content of dry matter (Method 925.09), crude ash (Method 923.03), crude protein (Method 920.87), ether extract (Method 920.39), and crude fiber (Method 962.09) with AOAC [22]. The content of crude protein (Method 920.87) and crude fiber (Method 962.09) in the feed mixes (*n* = 3) was determined using AOAC [22]. The metabolizable energy content was calculated as proposed by Janssen [28].

The Ca, Mg, P, Cu, Fe, Zn, K, and Na contents in the seeds and the Ca level in the mixtures (three replicates of each sample) were measured using flame atomic absorption spectrophotometry (FAAS) (Unicam 939/959AA-6300, Shimadzu Corp., Tokyo, Japan). Approximately 1 g of seeds or feed was weighed into heated porcelain crucibles. The samples were incinerated in a muffle furnace at 550 °C for 24 h, and then the ash was dissolved in 10 mL of 1 M HNO_3_. The following wavelengths were used for determination of elements: calcium at λ = 422.7 nm, magnesium at λ = 285.2 nm, copper at λ = 324.8 nm, iron at λ = 248.3 nm, zinc at λ = 213.9 nm, sodium at λ = 589 nm, and potassium at λ = 766.5 nm [31]. The total P content was determined according to the Polish standard PN-76/R-64781 [32] using a Helios Alpha UV-VIS spectrophotometer (Spectronic Unicam, Leeds, UK).

The content of anti-nutritional factors in the chickpea seeds was analyzed using the colorimetric method for determination of tannins [33], and the method described by Kakade et al. for assessment of the level of trypsin inhibitors [34]. 

The muscles (*n* = 20) were analyzed to determine the contents of dry matter (Method 925.09), crude ash (Method 923.03), crude protein (Method 920.87), and ether extract (Method 920.39) using AOAC [22]. The pH value was determined 24 h post slaughter using a Testo 205 m (Testo AG, Lenzkirch, Germany). The total cholesterol in the muscle samples was determined with the colorimetric method using an EPOLL 20 colorimeter and the C3045 standard (Sigma, Bellefonte, PA, USA) [35]. Color parameters (CIE L* a* b*) were assessed on the surface of freshly cut muscles using an 8200 reflection (X-Rite) spectrocolorimeter with illuminant D65 and a 10° standard observer [36]. In total, six measurements were made in each sample and the result was averaged for statistical analysis.

Fatty acids in the chickpea seeds, feed mixtures, and muscles were analyzed quantitatively and identified with the gas chromatography method using a Varian CP-3800 GC instrument (Varian, Harfsen, The Netherlands) and Supelco 37 FAME Mix 47885-U standards (Sigma, UK) after prior fat extraction with the diethyl ether-based Soxhlet method (Method 920.39) [22]. The procedure was performed after conversion of fats to fatty acid methyl esters (FAME) [37]. The technical and chemical details of the method for determination of fatty acids are described in a previous publication [28]. The content of individual fatty acids was expressed as a percentage of all identified fatty acids [38]. The values of meat dietary indicators, i.e., atherogenic indices (AI), thrombogenic indices (TI), and hypocholesterolemic/hypercholesterolemic ratio (h/H), were calculated using the following formulas:

AI = (C12:0 + 4 × C14:0 + C16:0)/(∑MUFA + ∑ (*n*−6) + ∑ (*n*−3)),

TI = (C14:0 + C16:0 + C18:0)/((0.5 × ∑MUFA + 0.5 × ∑ (*n*−6) + 3 × ∑ (*n*−3)) + (∑ (*n*−3)/∑ (*n*−6))),

based on [39], and

h/H = (C18:1 *n*−9 + C18:2 *n*−6 + C20:4 *n*−6 + C18:3 *n*−3 + C20:5 *n*−3 + C22:5 *n*−3 + C22:6 *n*−3)/(C14:0 + C16:0),

based on [40].

Before determination of the amino acid composition, the seed and feed samples were hydrolyzed in an aqueous solution (6N HCI + 0.5% phenol at 110 °C for 24 h), and analyzed with the use of ion exchange chromatography in an AAA 400 amino acid analyzer (Ingos Ltd., Praha, Czech Republic), as described in detail by Kwiecień et al. [41]. Cysteine and methionine were determined after oxidative hydrolysis [42].

#### 2.3.2. Lipid Profile in Serum and Antioxidant Status in Serum and Muscles

In the morning on day 42, blood of 10 cocks selected for slaughter (two cocks from each replicate) was collected from *vena cutanea ulnaris* into 6-mL lithium heparin-containing Vacutest tubes (Vacutest Kima s.r.l.) for determination of the lipid concentration and antioxidant status [28]. Plasma intended for the analysis of biochemical parameters was obtained by centrifugation of whole blood at 3000 rpm (603× *g*) for 15 min in a laboratory centrifuge (MPW-350R centrifuge, MPW Medical Instruments, Warsaw, Poland) at 4 °C, [28]. Cormay kits (PZ CORMAY SA, Łomianki, Poland) were used for determination of the content of triglycerides (TG), total cholesterol (TC), high-density lipoproteins (HDL), and low-density lipoproteins (LDL).

In the muscles and serum, the spectrophotometric method [43] was used for measurement of superoxide dismutase (SOD) activity at the wavelength of λ = 480 nm, whereas glutathione peroxidase (GPx) activity was determined at λ = 340 nm, with the use of ready-made Bioxytech GPx-340 tests (Oxis Research, Portland, OR, USA). Catalase activity (CAT) was determined with the colorimetric method proposed by Sinh [44] at the wavelength of λ = 570 nm. The biological material was additionally analyzed to detect the level of the lipid peroxidation product, i.e., malondialdehyde (MDA), with the method developed by Salih et al. [45]. The MDA results are expressed in nmol per 1 mg protein in the case of the meat, and in nmol per 1 mL in the case of the serum.

#### 2.3.3. Statistical Analysis

The data were analyzed using Statistica 12 software (StatSoft, Inc., Tulsa, OK, USA). The distribution of variables was tested for normality with the Shapiro–Wilk test. Normally distributed variables were compared using Student’s *t*-test. When the variables were not normally distributed, comparisons were made using the Mann–Whitney U test. The relationships between the parameters of the research material were also assessed using Pearson’s correlation coefficients. *p* < 0.05 was considered statistically significant in all tests.

## 3. Results

### 3.1. Productivity Parameters

The substitution of 50% of SBM with chickpeas as the main source of protein in the broiler chicken diets did not have a significant effect on the basic rearing parameters of the cocks, i.e., final body weight, IF, and FCR, on day 42 (Table 3).

### 3.2. The Chemical Composition, Color and pH Crude Breast and Thigh Meat

The substitution of SBM with CPR in the chicken diet had no significant effect on the pH values, which were higher in the breast muscles than in the thigh muscles (Table 4). The addition of CPR to the feed mixture contributed to a 5.5% increase in the lightness parameter (L*), an approximately 6% increase in crude ash content, and a 5.8% decline (*p* < 0.05) in the cholesterol content in the breast muscle measured 24 h after slaughter, compared to the SBM group (Table 4).

### 3.3. Fatty Acid Contents and Indices of Dietetic Value of Breast and Thigh Meat

Significant differences were observed in the content of individual fatty acids, especially in the breast muscle (Table 5). The differences in the content of *n*-3 and *n*-6 PUFAs were more pronounced in the CPR group. The content of these acids in the CPR group was significantly higher (by 42% and 11%, respectively) than in the SBM group. The total PUFA content was significantly increased (by 13%) in the CPR group, compared to SBM. There were differences between the groups in the total SFA and MUFA content: the CPR group was characterized by significantly lower levels of these acids (by 12% and 18.8%, respectively). The PUFA/SFA ratio increased (*p* < 0.05) by approximately 28% in the CPR group, compared to SBM. CPR was found to exert a significant effect on the dietary value of the breast meat (Table 5). The lowest (*p* < 0.05) values of S/*p*, *n*-6/*n*-3, AI, and TI were determined in the CPR group, compared to SBM (lower by 9%, 21.9%, 10%, and 13.9%, respectively). The CPR group was also characterized by a higher h/H ratio (by approximately 13%).

As in the case of the breast muscle, a decrease (*p* < 0.05) in the SFA and MUFA levels in the thigh muscle was observed in the CPR group (by 11% and 19%, respectively), in comparison with SBM (Table 6). The replacement of SBM at 310–350 g/kg diet chickpeas as the main protein source in the chicken feed resulted in a 30% increase in the PUFA_n-3_ content.

### 3.4. The Lipid Profile and Antioxidants in Serum

As shown by the results of the determination of the lipid profile (Table 7), there was no significant (*p* > 0.05) effect of CPR on TC, HDL-Chol, LDL-Chol, or TG, whereas the LDL/HDL ratio was significantly reduced by approximately 17%. No effect of the experimental factor on the parameters of antioxidant stress in the chicken serum was observed (Table 7).

### 3.5. Antioxidant Status in Meat

The experimental factor had no significant effect on the activity of the analyzed oxidative stress markers in the chicken breast and thigh muscles (Table 8).

### 3.6. Correlations between Lipid Profiles, Antioxidant Status, and Dietary Indices of Meat

The analysis of the correlation coefficients (r_2_) between the lipid profiles, antioxidant status, and dietary indices of the CPR-supplemented meat is presented in Table 9. A high negative correlation (r_2_ > −0.6) was determined in the following pairs: SFA-PUFA, Omg3, Omg6, h/H; MUFA-Omg3; PUFA-AI, TI; UFA-Omg3; Omg6-AI; AI-h/H; TI-h/H; CAT-GPx; GPx-TG in the breast muscle and SFA-PUFA, UFA, Omg6, h/H; PUFA-AI, TI; UFA-AI, TI; Omg3-TI; Omg6-AI, as well as AI-h/H and TI-h/H in the thigh muscle. Strong correlations (r_2_ > 0.6) were noted in the cases of SFA-AI, TI; MUFA-UFA; PUFA-Omg3, Omg6, h/H; Omg6- h/H; AI-TI; TC-LDL in the breast muscle and SFA-AI; PUFA-UFA, Omg6, h/H; UFA- Omg6, h/H; Omg6-h/H; AI-TI; SOD-MDA; and MDA-HDL in the thigh muscle. The values and trends in the correlation coefficients calculated for the parameters are probably related to the coexistence and co-involvement of other components in physiological and metabolic processes.

## 4. Discussion

A growing increase in the prices of cereals and protein concentrates has been observed in recent years. Intensive poultry production is based primarily on mixtures containing cereal grains and conventional protein sources, with SBM, often derived from GM varieties, as the most common component. The necessity of reduction of the impact of imported SBM, together with consumer disapproval of the use of GM feed in animal products, have inspired the search for alternative local sources of protein [46]. The chickpea (*Cicer arietinum* L.) appears to be a suitable plant to be used in Poland, as the quality of its protein is comparable to that of SBM [47]. However, there is a limited number of reports from Poland on the nutritional value of raw chickpea seeds and their nutritional effects in broiler chickens.

In the present study, the composition of the experimental diets met the nutritional requirements of broilers specified by the Aviagen [23]. The substitution of SBM from chickpeas at 310–350 g/kg diet as the main source of protein in the diets for the broiler chickens yielded similar production results. In the case of the SBM-fed chickens, the optimal production performance in the CPR group can be attributed to the well-balanced amino acid profile in combination with lysine, methionine, and threonine supplementation. The study results indicate that the replacement of SBM with 310–350 g/kg diet of CPR protein did not have a significant (*p* < 0.05) effect on the feed intake parameter. Since the mixtures were isoenergetic, it was expected that the birds would consume similar amounts of feed. However, the results of the experiments on partial replacement of SBM as a protein source with CPR are inconclusive. In the study conducted by Christodoulou et al. [24], partial replacement of SBM with raw chickpeas at a level of 120 kg/t of the concentrate mixture did not exert a negative effect on BW, DFC, or FCR, whereas an adverse effect was noted at the level of 240 kg/t of the concentrate mixture. The investigations reported by Viveros et al. [21] showed a negative effect of supplementation with 360 g⋅kg^−1^ of chickpea seeds on feed intake and conversion rates, as well as body weight gains. The discrepancies in the results may be caused by, for example, the level of supplementation, chickpea varieties, or the presence of anti-nutritional factors in the seeds, which may differ significantly between batches of the same legume material [48]. Nevertheless, compared to other legumes (e.g., soybeans), chickpeas contain relatively small amounts of inhibitors [49].

The color of meat is an important indicator of its quality, and one of the main attributes perceived by the customer, especially in boneless products. The present study demonstrated that the source of protein in the diet had an effect on the colorimetric indicators in the breast muscles. The breast muscles in the CPR group exhibited increased lightness, as evidenced by the higher L* value. This may be explained by the higher content of lipids in the breast muscle, which, in turn, may contain lower amounts of pigments, e.g., xanthophylls [50]. The meat color was within the normal range, and the muscles were not pale [51] The results of the present study also indicate a higher (*p* < 0.05) value of meat yellowness b* in the CPR-supplemented group compared to the SBM chickens, which can be explained by changes in the fatty acid profile and oxidative status (S/P) in the breast muscle [52].

An important parameter of meat quality is the cholesterol content, which can be modified through diet [53]. In this study, the breast meat of the CPR-fed chickens contained a lower level of cholesterol than the meat in the SBM group. This may be explained by the difference in the composition of the diet, and the presence of bioactive compounds in the chickpeas, which may influence cholesterol metabolism and alter cholesterol levels in chicken meat.

The quality of poultry meat depends on, for example, the content of dietary nutrients, stress experienced before slaughter, transport, genotype, sex, age [28,54], or the rearing system [55]. Modifications of the composition of dietary mixes can induce changes in the composition of meat, thus yielding a product with high nutritional value, providing substances that are beneficial to human health.

The content and ratio of omega-6 and omega-3 fatty acids are considered the main determinants of the h/H index. Therefore, due to their positive effects on health, they should be prioritized in broiler chicken feeding programs [56]. The recommended *n*-6/*n*-3 ratios range from 2-4:1 [57] and, together with the S/P ratio, they are common criteria of the nutritional value of fat [58]. Ayerza and Coates [59] found that supplementation with an alternative source of protein can significantly reduce the SFA content and the S/P and *n*-6/*n*-3 ratios in chicken meat, in comparison with the SBM diet, as in the case of the breast muscle analyzed in the present study. The CPR-supplemented diet also reduced the SFA level in the thigh muscle. The *n*-6/*n*-3 ratio in this experiment was 10:1, which is recommended for broiler meat, and consumption of such a product will not be detrimental to the FA profile and consumer health [60].

The present study showed that the differences in the h/H ratio mainly in the breast muscle of the broiler chickens were dependent on the protein source in the mixture. The breast muscles in the CPR group were characterized by not only a higher h/H ratio, but also by a lower total SFA level and higher contents of UFA/SFA, PUFA, omega-3, and omega-6, which is a favorable phenomenon. The CPR supplementation of the chicken diet was associated with a decline in the level of palmitic acid (C16:0), which represents SFAs similar to stearic acid. Both acids are well known for their hypercholesterolemic activity through elevation of serum and meat cholesterol levels [61]. Additionally, our study showed that the h/H ratio was highly correlated with TI; therefore, it is important to normalize the fatty acid content in animal diets in order to minimize the variability and improve the quality of chicken meat. As reported by Laudadio et al. [62], a high cholesterol level is correlated with high SFA content (r = −0.907; *p* = 0.001), which has not been confirmed in the present study. The differences in the TI and AI values and the content of omega-6 fatty acids provide further evidence for our hypothesis that the quality of broiler meat and its impact on human health may depend on the protein source. While omega-3 fatty acids play an important role in the regulation of TI, omega-6 acids have a dominant effect on the AI value [63]. The CPR-supplemented group exhibited better TI and AI values and higher content of omega-6 in the breast muscle. These differences can be attributed to the composition of the diet [64], and the results indicate that selective consumption of broiler meat can provide health benefits, i.e., not only higher intake of PUFAs, but also reduced TI and AI values, and an increased h/H ratio. Furthermore, it was observed that the breast muscle from the CPR-supplemented chickens was the richest source of linoleic acid. In turn, the meat from chickens from the SMB group had higher contents of eicosapentaenoic acid (EPA), which lowers total and cardiovascular mortality rates, and reduces the risk and development of tumors in animals [65]. The increase in the level of PUFAs observed in the present study was associated with the highest contents of not only linoleic acid (C18:2 *n*-6), but also eicosadienoic (20:2 *n*-6), arachidonic (20:4 *n*-6), and linolenic (C18:3 *n*-3) acids in the adipose tissue of the CPR-fed birds. Therefore, it can be concluded that CPR can replace the conventional SBM protein source with no adverse effects on production performance and meat quality. The positive effect of CPR was mainly observed in the chicken breast muscle.

As demonstrated by other studies, customers prefer poultry meat with high UFA content, due to its low levels of LDL/HDL cholesterol and lower AI values [66]. The application of an atherogenic diet (with higher contents of myristic and palmitic acid) is associated with an increase in total serum cholesterol, LDL and triglycerides, with a simultaneous reduction in the HDL concentration [67]. In the present study, there was no significant effect (*p* < 0.05) of CPR on the lipid profile in the blood of the chickens, with the exception of the favorable reduction in the LDL/HDL ratio. In addition, there are no reports on the effect of replacement of SBM protein with CPR protein on the antioxidant potential of blood and muscles in chicken; therefore, this study provides some knowledge of this issue. The absence of differences between the SBM and CPR groups may indicate that the addition of chickpeas to poultry feed does not elevate oxidative stress, and does not reduce meat quality. This associated with the presence of antioxidant substances in legume seeds, mainly such lipoxygenase inhibitors as isoflavones, phenolic acids, inositol phosphates, or tannins [68]. Typically, antioxidants are supplemented in sufficient amounts in diets to improve the stability of animal products, fatty acid profiles, and resistance [69].

## 5. Conclusions

The novelty of the present research is that it is the first study in Poland describing the effect of an alternative source of plant protein derived from native raw materials (CPR) on the serum antioxidant potential, lipid metabolism, fatty acid composition and profile, antioxidant status, and dietary values of breast and thigh muscles in broiler chickens. This nutritional study demonstrated that the substitution of the SBM protein with 310–350 g/kg diet of the CPR protein in the diet for chickens exerted no negative impact on the selected production performance parameters in these birds. Additionally, the use of chickpea seeds in the diet enriched the meat with PUFAs, especially the breast muscle, thus improving its nutritional value (favorable AI, TI, and h/H ratios). This offers an alternative to improve the quality and sales of chicken meat, in line with the global trend of consumption of functional food. Nevertheless, more research on different chickpea varieties is required in order to promote the use of this raw material in poultry diets.

## Figures and Tables

**Table 1:** Ingredients and chemical composition of experimental male broilers starter, grower, and finisher diets.

Item	Diets ^1^					
	Starter (0 to 21 Days)		Grower (21 to 35 Days)		Finisher (35 to 42 Days)	
	SBM	CPR	SBM	CPR	SBM	CPR
Diet composition, %						
Maize	20.00	20.00	23.26	10.00	25.65	10.00
Wheat	42.87	42.87	44.00	35.80	44.00	40.45
Soybean meal, 46% crude protein	29.95	29.95	24.94	13.00	22.98	11.50
Chickpea, 22.5% crude protein	-	-	-	34.41	-	31.51
Soybean oil	2.59	2.59	4.56	3.45	4.68	3.73
Dicalcium phosphate	1.47	1.47	0.96	1.00	0.74	0.77
Limestone	1.30	1.30	0.86	0.80	0.63	0.59
Na_2_SO_4_	0.23	0.23	0.17	0.11	0.16	0.10
L-Lys 78%	0.39	0.39	0.24	0.32	0.20	0.29
DL-Met 99%	0.35	0.35	0.25	0.26	0.21	0.22
L-Thr 99%	0.15	0.15	0.06	0.15	0.05	0.14
NaCl	0.20	0.20	0.20	0.20	0.20	0.20
Vitamin-mineral premix	0.50 ^2^	0.50 ^2^	0.50 ^3^	0.50 ^3^	0.50 ^4^	0.50 ^4^
Values calculated						
Metabolizable energy ^5^_,_ MJ⋅kg^−1^	12.4	12.4	13.2	13.1	13.3	13.3
Available P, %	0.470	0.470	0.350	0.350	0.299	0.300
Total Ca/Available P	2.02	2.02	2.00	2.00	2.00	2.00
Values analysed						
Crude protein, %	21.0	21.0	18.8	18.8	18.0	18.0
Crude fiber, %	2.84	2.84	2.73	2.27	2.70	2.28
Lysine, %	1.38	1.38	1.13	1.13	1.05	1.05
Methionine, %	0.668	0.668	0.544	0.549	0.494	0.498
Methionine + cysteine, %	1.03	1.03	0.879	0.879	0.821	0.819
Threonine, %	0.925	0.925	0.756	0.756	0.716	0.716
Total Ca, %	0.948	0.948	0.700	0.699	0.598	0.600
Fatty acids, g/100 g of total fatty acids						
Myristic (14:0)	0.500	0.510	0.470	0.480	0.480	0.470
Palmitic (16:0)	14.7	14.7	12.8	12.3	11. 7	12.0
Stearic (18:0)	4.31	4.31	3.69	3.64	3.50	3.30
Linoleic (18:2 *n*-6)	50.4	50.4	50.2	52.7	53.1	52.5
Linolenic (18:3 *n*-3)	4.73	4.73	4.47	5.06	6.43	5.35

^1^ SBM-100% protein comes from soybean meal, CPR-50% protein comes from soybean meal and 50% protein comes from raw chickpea; ^2^ Added minerals and vitamins per kg of starter diet: Mn 100 mg, I 1 mg, Fe 40 mg, Zn 100 mg, Se 0.15 mg, Cu 10 mg, vit. A 15,000 IU, vit. D3 5000 UI; vit. E 75 mg, vit. K3 4 mg, vit. B1 3 mg, vit. B2 8 mg, vit. B6 5 mg, vit. B12 0.016 mg, biotin 0.2 mg, folic acid 2 mg, nicotic acid 60 mg, pantothenic acid 18 mg, choline 1800 mg. ^3^ Added minerals and vitamins per kg of grower diet: Mn 100 mg, I 1 mg, Fe 40 mg, Zn 100 mg, Se 0.15 mg, Cu 10 mg, vit. A 12,000 IU, vit. D3 5000 UI, vit. E 50 mg, vit. K3 3 mg, vit. B1 2 mg, vit. B2 6 mg, vit. B6 4 mg, vit. B12 0.016 mg, biotin 0.2 mg, folic acid 1.75 mg, nicotic acid 60 mg, pantothenic acid 18 mg, choline 1600 mg; ^4^ Added minerals and vitamins per kg of finisher diet: Mn 100 mg, I 1 mg, Fe 40 mg, Zn 100 mg, Se 0.15 mg, Cu 10 mg, vit. A 12,000 IU, vit. D3 5000 UI, vit. E 50 mg, vit. K3 2 mg, vit. B1 2 mg, vit. B2 5 mg, vit. B6 3 mg, vit. B12 0.011 mg, biotin 0.05 mg, folic acid 1.5 mg, nicotic acid 35 mg, pantothenic acid 18 mg, choline 1600 mg; ^5^ Calculated according to European Table [28] as a sum of the metabolizable energy content of components.

**Table 2:** Chemical composition of raw chickpea.

Compounds	Chickpea
Basic nutrients, g⋅kg^−1^ dry matter	
Dry matter	911
Crude ash	28.5
Crude protein^1^	225
Ether extract	50.9
Crude fiber	16.4
Fatty acids, % of total fatty acids	
C14:0	0.20
C18:0	1.62
C20:0	0.71
C18:1	26.9
C18:2	55.0
C18:3	2.73
SFA	13.4
MUFA	27.5
PUFA	57.7
Minerals, fresh matter	
Ca, g	1.68
P, g	3.43
Mg, g	1.78
K, g	11.1
Na, g	0.780
Zn, mg	42.2
Cu, mg	10.7
Fe, mg	90.0
Amino acid composition, mg⋅g^−1^	
Histidine	4.06
Isoleucine	6.47
Leucine	12.5
Lysine	10.3
Methionine	3.40
Phenylalanine	8.78
Threonine	6.14
Tryptophan	6.52
Valine	6.57
Tyrosine	4.25
Arginine	11.9
Proline	7.36
Glycine	6.16
Alanine	6.94
Cysteine	3.66
Bioactive components, total in fresh matter	
Tannins, g⋅kg^−1^	0.281
Trypsin inhibitors, mg⋅g^−1^	4.80

Results are the average of 3 samples in three replicates; SFA-Saturated fatty acid, MUFA-Monounsaturated fatty acids; PUFA-Polyunsaturated fatty acids. ^1^ Calculated by Kjeldahla nitrogen N × 6.25.

**Table 3:** Effect of substitution of soybean meal with raw chickpea seeds on selected growth performance parameters of male broilers.

Item	SBM	CPR	SEM	*p*-Value
Final BW (g)	2031	2059	14.5	0.363
Total IF (g/bird)	3265	3013	0.11	0.796
FCR, (kg/kg)	1.93	1.88	71.7	0.075
Daily BWG, g bird	54.0	53.0	0.18	0.276
Mortality of chicken, heads	0.00	3.00	-	-

Data represent the mean of 10 broiler chickens per treatment; SBM—100% protein comes from soybean meal; CPR—50% protein comes from soybean meal and 50% protein comes from raw chickpea; BW—body weight; SEM—standard error of the mean; IF—feed intake; FCR—feed conversion ratio; BWG—body weight gain.

**Table 4:** pH, moisture, color, and chemical composition of crude breast and thigh meat of male broilers.

Item	SBM	CPR	SEM	*p*-Value
Breast meat				
pH_24_	5.37	5.41	0.243	0.059
Moisture (%)	75.8	76.8	0.281	0.112
L* (lightness)	50.4^b^	53.2 ^a^	0.861	0.036
a* (redness)	8.78	9.05	0.781	0.091
b* (yellowness)	1.91 ^b^	2.15 ^a^	0.041	0.045
Dry matter	24.3	23.3	0.244	0.051
Crude fat	1.33	1.31	0.035	0.816
Crude protein	21.9	21.0	0.217	0.052
Crude ash	1.14 ^b^	1.21 ^a^	0.016	0.041
Cholesterol, mg⋅100 g^−1^	45.7 ^b^	43.2 ^a^	1.896	0.042
Thigh meat				
pH_24_	5.28	5.33	0.231	0.054
Moisture (%)	74.9	74.8	0.721	0.322
L* (lightness)	49.2	47.9	0.785	0.067
a* (redness)	10.9	10.3	0.642	0.221
b* (yellowness)	1.67	1.61	0.541	0.081
Dry matter	25.1	25.2	0.316	0.062
Crude fat	7.26	6.97	0.256	0.051
Crude protein	17.4	17.4	0.085	0.787
Crude ash	1.04	1.03	0.018	0.834
Cholesterol, mg⋅100 g^−1^	40.5	39.7	0.036	0.087

Data represent the mean of 10 broiler chickens per treatment. SBM—100% protein comes from soybean meal; CPR—50% protein comes from soybean meal and 50% protein comes from raw chickpea; ^a,b^—mean values in rows with different letters differ significantly at *p* < 0.05.

**Table 5:** Fatty acid profile (g/100 g of total fatty acids) in male broilers breast meat.

Item	SBM	CPR	SEM	*p*-Value
SFA				
Myristic acid (C14:0)	0.458	0.476	0.021	0.698
Pentadecanoic acid (15:0)	0.086	0.114	0.009	0.064
Palmitic acid (16:0)	21.1 ^a^	17.8 ^b^	0.595	0.011
Heptadecanoic acid (17:0)	0.092 ^a^	0.118 ^b^	0.010	0.042
Stearic acid (18:0)	7.01	6.85	0.268	0.786
Arachidic acid (20:0)	0.066 ^b^	0.082 ^a^	0.005	0.031
Behenic acid (22:0)	0.032 ^b^	0.040 ^a^	0.003	0.017
MUFA				
Margaroleic acid (17:1)	0.022 ^b^	0.048 ^a^	0.006	0.024
Oleic acid (18:1)	40.5 ^a^	32.9 ^b^	1.836	0.028
Eicosenoic acid (20:1)	0.416 ^a^	0.364 ^b^	0.013	0.026
PUFA				
Linoleic acid (18:2_*n*-6_)	31.0 ^b^	33.9 ^a^	0.786	0.048
Eicosadienoic acid (20:2_*n*-6_)	0.224 ^b^	0.426 ^a^	0.036	0.001
Arachidonic acid (20:4_*n*-6_)	1.49 ^b^	1.97 ^a^	0.125	0.041
Alfa-linoleic acid (18:3_*n*-3_)	2.40 ^b^	3.41 ^a^	0.212	0.006
Eicosapentaenoic acid (20:5_*n*-3_)	0.096 ^a^	0.071 ^b^	0.006	0.029
∑SFA	28.9 ^a^	25.5 ^b^	0.607	0.044
∑MUFA	40.9 ^a^	33.2 ^b^	1.854	0.028
∑PUFA	35.2 ^b^	39.8 ^a^	0.988	0.009
∑UFA	76.1	73.0	1.617	0.374
∑PUFA*_n_*_-6_	32.7 ^b^	36.3 ^a^	0.863	0.027
∑PUFA*_n_*_-3_	2.40 ^b^	3.41 ^a^	0.212	0.006
∑PUFA/∑SFA	1.22^b^	1.56 ^a^	0.064	0.004
∑SFA/∑PUFA	0.82	0.70	0.019	0.125
∑UFA/∑SFA	2.46 ^b^	2.87 ^a^	0.091	0.031
Indices of the dietary value of meat				
∑S/P	0.377 ^a^	0.346 ^b^	0.009	0.025
*n*-6/*n*-3 ^1^	13.6 ^a^	10.7 ^b^	0.740	0.041
AI	0.303 ^a^	0.270 ^b^	0.006	0.012
TI	0.65 ^a^	0.56 ^b^	0.019	0.024
h/H	3.49 ^b^	3.96 ^a^	0.097	0.016

Data represent the mean of 10 broiler chickens per treatment. SBM—100% protein comes from soybean meal; CPR—50% protein comes from soybean meal and 50% protein comes from raw chickpea; SEM—standard error of the mean; SFA—saturated fatty acids; MUFA—monounsaturated fatty acids; PUFA—polyunsaturated fatty acids; UFA—unsaturated fatty acids; S/P—ratio of saturated fatty acids to unsaturated fatty acids; ^1^ *n*-6/*n*-3—the calculated *n*-6/*n*-3 ratio was a sum of [(C18:2 *n*-6, C20:2 *n*-6, C20:4 *n*-6)/(C18:3 *n*-3, 20:3 *n*-3)]; AI—atherogenic index; TI—thrombogenic index; h/H—hypocholesterolemic/Hypercholesterolemic ratio; ^a,b^—means in the same rows with different letters differ significantly at *p* < 0.05.

**Table 6:** Fatty acid profile (g/100 g of total fatty acids) in male broilers thigh meat.

Items	SBM	CPR	SEM	*p*-Value
SFA				
Myristic acid (C14:0)	0.410	0.516	0.045	0.263
Pentadecanoic acid (15:0)	0.080	0.104	0.008	0.149
Palmitic acid (16:0)	21.2 ^a^	18.8 ^b^	0.423	0.0001
Heptadecanoic acid (17:0)	0.130	0.144	0.013	0.622
Stearic acid (18:0)	5.97	6.02	0.141	0.871
Arachidic acid (20:0)	0.070	0.080	0.007	0.091
Behenic acid (22:0)	0.018	0.020	0.003	0.740
MUFA				
Margaroleic acid (17:1)	0.291 ^a^	0.193 ^b^	0.019	0.001
Oleic acid (18:1)	0.070	0.088	0.008	0.282
Eicosenoic acid (20:1)	0.486	0.425	0.029	0.284
PUFA				
Linoleic acid (18:2_*n*-6_)	25.6	26.5	2.114	0.856
Eicosadienoic acid (20:2*_n_*_-6_)	0.184	0.228	0.019	0.270
Arachidonic acid (20:4*_n_*_-6_)	0.296 ^b^	0.574 ^a^	0.052	0.001
Alfa-linoleic acid (18:3*_n_*_-3_)	2.34 ^b^	3.06 ^a^	0.143	0.003
Eicosapentaenoic acid (20:5*_n_*_-3_)	0.058	0.060	0.003	0.792
∑SFA	29.5 ^a^	26.5 ^b^	0.684	0.002
∑MUFA	41.9 ^a^	35.2 ^b^	1.509	0.001
∑PUFA	28.5	30.4	2.181	0.691
∑UFA	70.5	65.6	1.735	0.176
∑PUFA_*n*-6_	26.1	27.3	2.129	0.803
∑PUFA_*n*-3_	2.34 ^b^	3.06 ^a^	0.143	0.002
∑PUFA/∑SFA	0.966	1.16	0.092	0.374
∑SFA/∑PUFA	0.39	0.38	0.010	0.682
∑UFA/∑SFA	2.66 ^a^	2.37 ^b^	0.024	0.042
Indices of the dietary value of meat				
∑S/P	0.395	0.386	0.010	0.682
*n*-6/*n*-3	11.2	8.87	0.874	0.209
AI	0.347	0.329	0.016	0.609
TI	0.675	0.626	0.017	0.151
h/H	3.22	3.37	0.077	0.376

Data represent the mean of 10 broiler chickens per treatment. SBM—100% protein comes from soybean meal; CPR—50% protein comes from soybean meal and 50% protein comes from raw chickpea; SEM—standard error of the mean; SFA—saturated fatty acids; MUFA—monounsaturated fatty acids; PUFA—polyunsaturated fatty acids; UFA—unsaturated fatty acids; S/P—ratio of saturated fatty acids to unsaturated fatty acids; *n*-6/*n*-3—the calculated *n*-6/*n*-3 ratio was a sum of [(C18:2 *n*-6, C20:2 *n*-6, C20:4 *n*-6)/(C18:3 *n*-3, 20:3 n-3)]; AI—atherogenic index; TI—thrombogenic index; h/H—hypocholesterolemic/Hypercholesterolemic ratio; ^a,b^—means in the same rows with different letters differ significantly at *p* < 0.05.

**Table 7:** Lipid concentration (mmol·L^−1^) and antioxidant enzyme in male broilers’ plasma.

Item	SBM	CPR	SEM	*p*-Value
TC, mg⋅dL^−1^	3.36	3.24	0.084	0.523
HDL-Chol, mg⋅dL^−1^	2.36	2.48	0.062	0.381
LDL-Chol, mg⋅dL^−1^	0.504	0.435	0.078	0.152
TG, mg⋅dL^−1^	0.822	0.816	0.023	0.113
LDL/HDL	0.214 ^a^	0.176 ^b^	0.058	0.043
SOD, U⋅mg^−1^ of protein	40.5	42.2	2.108	0.065
CAT, U⋅mg^−1^ of protein	53.0	52.8	1.023	0.095
GPx, U⋅mg^−1^ of protein	5.22	4.98	0.092	0.073
MDA, nmol⋅mL^−1^ of serum	47.1	46.0	23.3	0.062

Data represent the mean of 10 broiler chickens per treatment. SBM—100% protein comes from soybean meal; CPR—50% protein comes from soybean meal and 50% protein comes from raw chickpea; TC—total cholesterol; TG—total triglyceride; HDL—high density lipoprotein; LDL—low density lipoprotein; SOD—superoxide dismutase; CAT—catalase; GPx—glutathione peroxidase; MDA—malondialdehyde; SEM—standard error of the mean; ^a,b^—means in the same rows with different letters differ significantly at *p* < 0.05.

**Table 8:** Antioxidant enzyme activity in crude male broilers’ breast and thigh meat.

Item	SBM	CPR	SEM	*p*-Value
Breast meat				
SOD, U⋅g^−1^ protein	41.2	41.0	1.901	0.184
CAT, U⋅mg^−1^ protein	0.308	0.311	0.012	0.343
GPx, U⋅mg^−1^ protein	2584	2577	33.3	0.421
MDA, nmol⋅mg^−1^ protein	1.47	1.48	0.213	0.112
Thigh meat				
SOD, U⋅g^−1^ protein	40.2	40.3	0.998	0.223
CAT, U⋅mg^−1^ protein	0.274	0.283	0.007	0.082
GPx, U⋅mg^−1^ protein	2556	2571	45.2	0.218
MDA, nmol⋅mg^−1^ protein	1.45	1.46	0.125	0.331

Data represent the mean of 10 broiler chickens per treatment; SBM—100% protein comes from soybean meal; CPR—50% protein comes from soybean meal and 50% protein comes from raw chickpea; SEM—standard error of the mean; SOD—superoxide dismutase; CAT—catalase; GPx—glutathione peroxidase; MDA—malondialdehyde.

**Table 9:** Correlation coefficients (r_2_) between lipid profiles, antioxidant status, and indicators of nutritional value in the breast and thigh muscles of broiler chickens; significant values at *p* < 0.05.

	SFA	MUFA	PUFA	UFA	Omg3	Omg6	AI	TI	h/H	SOD	CAT	GPx	TC	HDL	LDL
Breast meat															
MUFA	ns														
PUFA	−0.816 **	ns													
UFA	ns	0.847 **	ns												
Omg 3	−0.810 **	−0.912 **	0.662 *	−0.642 *											
Omg 6	−0.740 *	ns	0.984 **	ns	ns										
AI	0.898 **	ns	−0.841 **	ns	ns	−0.836 **									
TI	0.923 **	ns	−0.888 **	ns	−0,617	−0.866 **	0.943 **								
h/H	−0.860 **	ns	0.784 **	ns	ns	0.789 **	−0.993 **	−0.920 **							
SOD	ns	ns	ns	ns	ns	ns	ns	ns	ns						
CAT	ns	ns	ns	ns	ns	ns	ns	ns	ns	ns					
GPx	ns	ns	ns	ns	ns	ns	ns	ns	ns	ns	−0.612				
TC	ns	ns	ns	ns	ns	ns	ns	ns	ns	ns	ns	ns			
HDL	ns	ns	ns	ns	ns	ns	ns	ns	ns	ns	ns	ns	ns		
LDL	ns	ns	ns	ns	ns	ns	ns	ns	ns	ns	ns	ns	0.732 *	−0,296	
*TG*	ns	ns	ns	ns	ns	ns	ns	ns	ns	ns	ns	−0.659 *	ns	−0.277	0.167
Thigh meat															
	SFA	MUFA	PUFA	UFA	Omg3	Omg6	AI	TI	h/H	SOD	CAT	GPx	TC	HDL	LDL
MUFA	0.397														
PUFA	−0.880 **	ns													
UFA	−0.700 *	ns	0.728 *												
Omg 3	ns	ns	ns	ns											
Omg 6	−0.867 **	ns	0.999 **	0.714 *	ns										
AI	0.893 **	ns	−0.933 **	−0.780 **	ns	−0.926 **									
TI	ns	ns	−0.626	−0.849 **	−0.686 *	ns	0.776 **								
h/H	−0.649 *	ns	0.750 *	0.877 **	ns	0.741 *	−0.872 **	−0.917 **							
SOD	ns	ns	ns	ns	ns	ns	ns	ns	ns						
CAT	ns	ns	ns	ns	ns	ns	ns	ns	ns	ns					
GPx	ns	ns	ns	ns	ns	ns	ns	ns	ns	ns	ns				
TC	ns	ns	ns	ns	ns	ns	ns	ns	ns	ns	ns	ns			
HDL	ns	ns	ns	ns	ns	ns	ns	ns	ns	ns	ns	ns	ns		
LDL	ns	ns	ns	ns	ns	ns	ns	ns	ns	ns	ns	ns	0.732 *	−0.296	
TG	ns	ns	ns	ns	ns	ns	ns	ns	ns	ns	ns	ns	ns	−0.277	0.167

MUFA—monounsaturated fatty acids; PUFA—polyunsaturated fatty acids; UFA—unsaturated fatty acids; Omg 3—omega *n*—3 fatty acids; Omg 6—omega *n*-6 fatty acids; AI—atherogenic index; TI—thrombogenic index; h/H—hypocholesterolemic/Hypercholesterolemic ratio; SOD—superoxide dismutase; CAT—catalase; GPx—glutathione peroxidase; TC—total cholesterol; TG—total triglyceride; HDL—high density lipoprotein; LDL—low density lipoprotein. **—correlation significant at 0.01 level; *—correlation significant at 0.05 level.

## Data Availability

The data presented in this study are available on request from the corresponding author.
